# Constitutively active orphan G protein-coupled receptors through the lenses of cryo-electron microscopy

**DOI:** 10.1016/j.jbc.2025.110571

**Published:** 2025-08-08

**Authors:** Josep Argerich, Daniel Muñoz-Reyes, Iris del Val-García, Javier García-Nafría

**Affiliations:** Institute for Biocomputation and Physics of Complex Systems (BIFI) and Laboratorio de Microscopías Avanzadas (LMA), University of Zaragoza, Zaragoza, Spain

**Keywords:** orphan GPCRs, constitutively active GPCR, in-built agonists, lipid signaling, G protein-coupled receptor (GPCR), cryo-electron microscopy, cell signaling, receptor structure-function

## Abstract

G protein-coupled receptors (GPCRs) are a therapeutically privileged family of receptors involved in a wide variety of pathophysiological conditions and the successful target for ∼34% of FDA-approved drugs. However, a significant percentage of GPCRs remain orphan, *i.e.*, the endogenous ligands that modulate receptor function are unknown, and hence knowledge about their functional role and the generation of new therapeutics lag behind. During recent years, the use of cryo-electron microscopy has revolutionized GPCR structural biology including its application to orphan GPCRs, especially those displaying constitutive activity in cellular model systems. Such efforts have resulted in the description of new modes of in-built agonists that include the ECL2 and N-terminal regions as well as identifying ubiquitous endogenous ligands readily bound to GPCRs. These results position structural determination as a new key component in GPCR deorphanization, shedding light on new signaling mechanisms, bringing questions about their functional regulation, and opening new avenues for drug design.

G protein-coupled receptors (GPCRs) are a family of membrane proteins that mediate intercellular communication across major body organs as well as being the target of ∼34% of FDA-approved drugs (1, 2). GPCRs are generally located at the plasma membrane and sense a wide variety of extracellular chemical and physical stimuli (including photons, ions, neurotransmitters, hormones and peptides) through a shared seven-transmembrane fold that, once activated, populates active-state conformations that couple to and activate heterotrimeric Gαβγ proteins and β-arrestins (3). There are ∼800 GPCRs that are classified into different families including Class A (Rhodopsin-like family, which accounts for >80% of all GPCRs), Class B (Secretin-like family), Class C (Metabotropic glutamate receptors), Adhesion, Class D (Pheromone receptors), Class E (cAMP receptors), and Class F (Frizzled/smoothened family). Out of the ∼360 non-olfactory human GPCRs, there are still over 90 for which there is no identified (or agreed by the scientific community) endogenous agonist and hence do not have a clearly defined function - these are termed orphan GPCRs (oGPCRs hereafter). The majority of oGPCRs are class A receptors (with 83 members listed in the International Union of Pharmacology as of April 2025, while there are 8 Class C and none for Class B) (4). After the initial identification of the GPCR family members in the human genome (5, 6, 7) and the creation of the IUPHAR database (8), there was a spike in studies proposing new pairings between oGPCRs and cognate ligands. This was achieved through reverse pharmacology, where known receptors were screened against known transmitters as well as the screening of tissue extracts (9). However, a decrease in the pace of receptor-ligand pairings has been appreciated since 2006, likely highlighting that low hanging fruits have been already “grasped” and non-trivial deorphanization campaigns are left (10, 11). Several challenges pose barriers to further oGPCR deorphanization including (i) the potential production of endogenous molecules only in specific cell types and/or under specific circumstances, therefore requiring the generation of specific spatiotemporal conditions which are difficult to reproduce or even identify, (ii) the potential requirement for co-receptors or additional novel transducers present in specific cell types, (iii) the potential antagonistic role of endogenous modulators, which are likely missed by screening campaigns using activation-reporting assays, and (iv) the fact that receptors might have ligand-independent functions such as being constitutively active (12, 13, 14) or acting as auxiliary subunit/regulator of additional receptors (15, 16). Additionally, irreproducibility between laboratories is a current challenge (11), likely because of single-assay artifacts or the use of different cell models that provide (or not) the oGPCRs with the necessary elements to carry out their signaling function. Such issues highlight the need for new approaches to tackle receptor deorphanization.

Cryo-electron microscopy (cryo-EM) has revolutionized structural biology and even more that of GPCRs. As of April 2025, the structure of 238 unique GPCRs have been determined, with cryo-EM playing an increasingly important role, as shown in the GPCRdb (17). Cryo-EM is playing a key role in understanding GPCR activation, Gαβγ protein and β-arrestin coupling (18, 19), drug binding modes (examples of, but not limited to (20, 21)), oligomerization (18, 22, 23), or biased agonism (examples of, but not limited to (24, 25, 26, 27)). In recent years, the application of cryo-EM to oGPCRs has yielded structures of 23 new orphan GPCRs. Such efforts have uncovered the ways these receptors recognize their proposed ligands (28), how they activate upon sensing of environmental conditions, such as extracellular pH (29), as well as describing new signaling reactions (30). In this review, we focus on recent insights about where cryo-EM has been key to GPCR deorphanization. This includes (i) the identification of unexpected densities in oGPCR structures, which triggered the identification of the cognate ligand and (ii) the identification of new mechanisms that drive constitutive activity in oGPCRs. Additional unassigned densities identified in oGPCRs structures still exist and require further identification through experimental or structure-based computational approaches. Hence, structural determination is becoming a key tool in the understanding of oGPCRs and their deorphanization.

## Constitutive activity in oGPCRs

Canonical GPCRs are sensors for physical or chemical stimuli, where a molecule on the extracellular space binds to a pocket at the extracellular region of the receptor. This recognition triggers receptor activation, occurring through a conformational change, which is then able to bind and activate heterotrimeric Gαβγ proteins and β-arrestins in the intracellular milieu. Activation of transducers triggers signaling cascades that yield in a cell-specific response (3, 31). In contrast, constitutive activity is the spontaneous activation of transducer signaling (*e.g.* Gαβγ activation) by the receptor in the absence of agonist (32). Some degree of constitutive activity is always present in most GPCRs and such constitutive activity has been used to define G protein signaling pathways in oGPCRs when no known agonists were available (12, 32, 33). However, herein, we will discuss constitutively active receptors with an apparent ligand-independent activity comparable in magnitude to classical receptors activated with agonists.

Technically, constitutive activity is measured using *in cellulo* assays, where cDNAs for the receptor and the specific Gαβγ proteins are transiently transfected. A wide range of readouts are available and include monitoring the activation/dissociation of Gαβγ heterotrimers by Bioluminescence Resonance Energy Transfer (BRET2) (34), recruitment of Gαβγ (35), the accumulation of downstream cAMP second messenger (by *e.g.* HTRF or Homogeneous Time Resolved Fluorescence), transcription-based assays such as Presto-Tango (36) or GTP turnover assays (37). Additional modifications with enhanced sensitivity and reduced noise levels have been proposed and suggested to be especially suitable for detecting GPCR basal activities (12, 38). Strategies to detect and quantify basal activities include transfecting increasing amounts of the receptor cDNA (39) and/or comparing the basal activity with an established ligand-activated receptor in the presence and absence of its endogenous modulator, or with a negative control such as receptors lacking constitutive activity or cells transfected with mock cDNA. Care must be taken when comparing the constitutive activity of different receptors since, depending on the assay, the signal might not be comparable. This includes comparing events that occur near the receptor (*e.g.* Gαβγ dissociation) and downstream signaling events (*e.g.* cAMP accumulation) where the signal suffers amplification or comparing the dissociation of different Gα proteins by BRET assays, where the efficacy varies with the type of Gα due to differences in BRET efficiency.

Systematic studies using various functional assays in oGPCRs noted that a great proportion of these receptors displayed high constitutive activity in cellular models (12, 13, 14), suggesting either the presence of intrinsic constitutive activity mechanisms, or the presence of ubiquitous endogenous ligands. Mechanistically, GPCRs have been shown to achieve intrinsic constitutive activity by piling up a set of interactions that collectively enhance their basal activity (40, 41), seen as well in genetic variations that enhance constitutive activity of GPCRs in disease states (14, 42, 43, 44). Additionally, GPCRs can have an in-built agonist motif, where part of the receptor itself penetrates the orthosteric binding pocket to mimic an agonist, thus activating the receptor. The presence of regulated in-built agonists is found in the Stachel sequence of Adhesion GPCRs (45, 46, 47) as well as in protease-activated receptors (48, 49). Such sequences are found at the N-terminus and are normally kept away from the agonist binding site by being embedded as part of a larger protein domain that sterically impedes its binding into the orthosteric binding pocket. Upon regulated N-terminal proteolytic cleavage the tethered agonist is free to activate the receptor.

Recently, cryo-EM structural determination of “macroscopically” (macroscopically referring to the intrinsic activity as observed in cellular assays) constitutively active oGPCRs (caoGPCRs hereafter) has uncovered new modalities of in-built agonists where different modes of Extracellular Loop 2 (ECL2) and N-terminal engagement at the orthosteric binding site trigger receptor activation. Additionally, it has also been uncovered that some of these receptors are not constitutively active, but rather constitutively activated by the presence of an ubiquitous agonist that constantly triggers receptor activation. These two scenarios will be discussed separately.

## New in-built agonists in constitutively active oGPCRs

### New in-built agonist at the N-terminus of GPCRs

**Figure 1:**
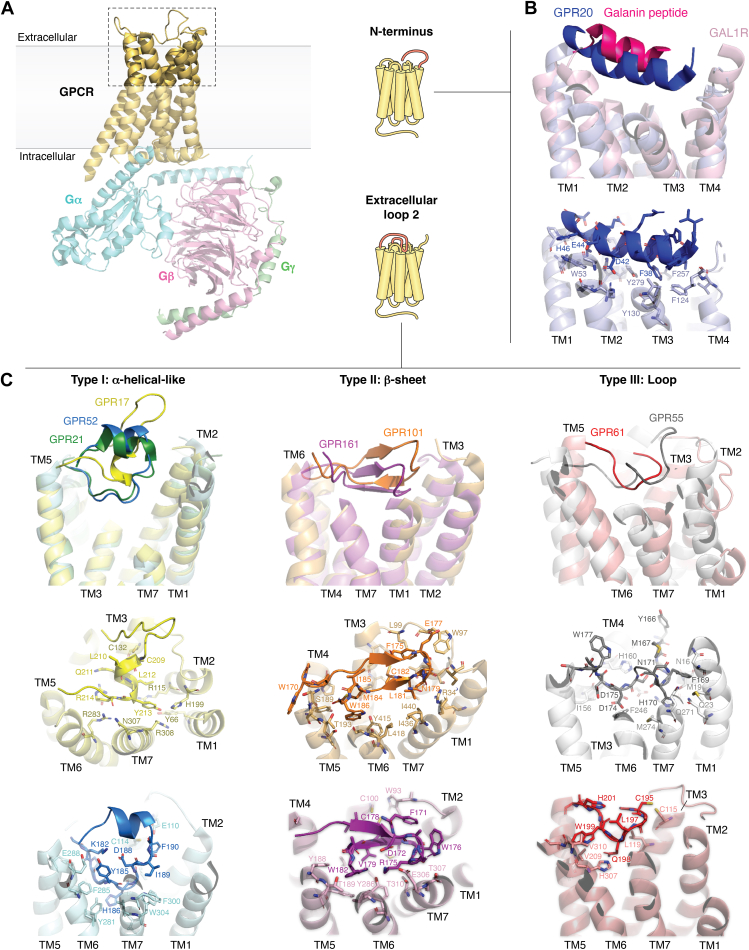
**Constitutively active receptors with an in-built agonist.***A*, representative structure of a GPCR (PDB: 9F33) in complex with a G protein heterotrimer. The region highlighted with a *dashed box* is magnified in *panels* (*B*) and (*C*). *B*, structural superposition of GPR20 (PDB: 8HSC) and the galanin receptor 1 (GAL1R) bound to the galanin peptide (PDB: 7WQ3). Structures are shown as transparent cartoons, except for the N-terminus and the galanin peptide, which are displayed in strong colors. *C*, classification of GPCRs with in-built agonists located in extracellular loop 2 (ECL2), based on ECL2 conformation. Receptors are displayed as transparent cartoons, with ECL2 in strong colors. *Left panel*: superposition of GPR17 (PDB: 7Y89), GPR21 (PDB: 8HJ1), and GPR52 (PDB: 6LI3). Center panel: superposition of GPR101 (PDB: 8W8Q) and GPR161 (PDB: 8KH4). *Right panel*: superposition of GPR55 (PDB: 9IY8) and GPR61 (PDB: 8TB0). All structures are shown as cartoons and colored by receptors.

Tethered agonism in GPCRs is usually associated with having an in-built agonist at the N-terminus, albeit always occurring in a regulated manner in adhesion GPCRs and protease-activated receptors (45, 46, 47, 48, 49). More recently, new manners in which the N-terminus could act as an in-built agonist were reported. GPR20 is a receptor with unknown function but significant expression in intestine and highly expressed in gastrointestinal stromal tumors (50). GPR20 was shown to display high constitutive activity and, as an example, it was shown to have similar activity to that of the apelin receptor activated with an agonist, as measured monitoring Gαβγ dissociation in HEK293 cells using BRET2 assays (34, 51). Structural determination of GPR20 showed the presence of a unique N-terminal cap, in the form of an α-helix that binds deep into the pocket of the seven-transmembrane fold with a phenylalanine residue (F38^N-term^) packing deep in the pocket with aromatic π-π stacking with Y130^3.32^, F257^6.51^ and M134^3.36^ and being key for the receptor activity, since its mutation reduced the constitutive activity by 50%. In this case, the ECL2 is short and flexible, and lacks class A conserved disulfide bonds, likely allowing for the N-terminal cap to have a prominent functional role. N-terminal α-helical caps are typical of lipid receptors, and they can be seen on top of the seven-transmembrane fold in sphingosine-1-phosphate receptor or the lysophosphatidic acid receptor (52). However, in contrast to lipid receptors, the N-terminal α-helix in GPR20 is buried deeply into the pocket (Fig. 1*B*), and in a similar manner than the galanin peptide when acting as an agonist in galanin receptors (Fig. 1*B*). Although there is no (known) functional resemblance between GPR20 and galanin receptors, it seems such α-helical binding mode at the orthosteric pocket is common between agonist peptides and in-built agonists.

### ECL2 as a common in-built agonist in caoGPCRs

The first structure of a caoGPCR with an ECL2 in-built agonist was that of GPR52, where the ECL2 protrudes into the orthosteric binding site activating the receptor (53). This was followed by structures of GPR17 (54), GPR21 (55, 56), GPR101 (57), GPR61 (14, 58), GPR55 (59, 60, 61) and GPR161 (14, 58). All seven receptors contain an ECL2 penetrating into the orthosteric binding pocket, some of which are positioned at equivalent sites of classical endogenous agonists. Based on the conformation of the ECL2 that dives into the pocket, different types of in-built ECL2 agonists can be distinguished, which we classify in (i) Type I, α-helical-like conformation (GPR52, GPR21, GPR17), (ii) Type II, β-sheet conformation (GPR161, GPR101), and (iii) Type III, loop conformation (GPR55, GPR61) (Fig. 1).

Type I ECL2 includes GPR52, GPR21 and GPR17 which display an α-helical ECL2 that inserts in the orthosteric binding site of the receptors. GPR52 and GPR21 signal mainly through Gα_S_ and share phylogenetic relationship (71% sequence identity), although they seem to have different physiological roles. While GPR52 is highly expressed in human striatal brain and is a promising target for schizophrenia and other neurological disorders (62), GPR21 is expressed in the brain and spleen and regulates body weight and glucose metabolism (63, 64). GPR17 has no sequence similarity to GPR52 or GPR21 and instead has been grouped with purine P2Y receptors and cysteinyl-leukotriene receptors (65). It is known to express in oligodendrocyte precursor cells and regulate their differentiation and maturation (66). Additionally, it has been proposed to be a marker for neuronal injury and a potential target for multiple sclerosis (66, 67). Structurally, the ECL2 of GPR52/GPR21 superpose closely and show an N-terminal activating α-helix (a loop in some structures that orders into an α-helix upon ligand binding) followed by an extracellular C-terminal α-helix, which acts as a lid for the orthosteric binding site entry (Fig. 1*C*). The key role of the in-built agonist has been shown by either replacing the activating segment of the ECL2 with a linker (loosing 75% (BRET2) and 90% (cAMP) activity for GPR21 and GPR52 respectively) or mutating C^45.50^ (which forms a disulfide bridge with C^3.25^) and a lysine residue (K170^ECL2^E and K182^ECL2^E in GPR21 and GPR52 respectively), two mutations that disrupt the structure of the ECL2. Opposite to GPR52/GPR21, the ECL2 in GPR17 displays an N-terminal extracellular hairpin-like structure followed by an activating C-terminal α-helix-like structure (Fig. 1*C*). Similar to GPR52/21, replacing the ECL2 activating C-terminal segment of GPR17 with a GGSGGS linker or mutating Y213^ECL2^ yielded a significant reduction in activity as measured with cAMP assays. Major differences between the two sets include the position of the activating segment within the ECL2 (N-terminal for GPR52/GPR21 and C-terminal for GPR17) and that the activating α-helix inserts into the binding pocket perpendicular to the membrane in the case of GPR17, while it inserts at an angle more parallel to the plane of the membrane in the case of GPR52/GPR21. The ECL2 in the three receptors forms interactions with TM6 and TM7; however, the ECL2 in GPR17 makes interactions with TM2 while GPR52/GPR21 do not (Fig. 1*C*).

Type II ECL2 is found in GPR101 and GPR161, where an antiparallel β-sheet protrudes into the ligand binding pocket (Fig. 1*C*). GPR101 is expressed in the nucleus accumbens and hypothalamus (68), and controls energy homeostasis and the production of growth hormone (69), while GPR161 is located in cilia and is best known for its negative regulation of the Sonic hedgehog pathway (70). Overall, a β-sheet at the ECL2 has been associated most commonly in peptide and prostanoid receptors (71, 72); however, in this case, this structure seems to have diverged to form an in-built agonist. The antiparallel β-sheet in both receptors adopt a similar conformation, with the C-terminal β-strand (closer to TM5) protruding into the ligand-binding pocket, and the most N-terminal β-strand (closer to TM4) being exposed to the solvent (Fig. 1*C*). The β-sheet ECL2 in GPR101 has a shallower position when compared to that in GPR161, specially towards the β-turn next to TM1-TM2-TM7 where there is a 5 Å displacement between main chain atoms. Within the ECL2 there seems to be β-sheet anchoring interactions (of varying nature between the two receptors but share location at the β-turn and form interactions mainly with ECL1 of the receptor) and receptor activating interactions (which pack deeper into the core), although it is not possible to differentiate activating and anchoring interactions relying exclusively of mutagenesis and functional assays. The most structurally and sequence conserved positions between the two receptor ECL2s is a conserved cysteine (C104 and C100 in GPR101 and GPR161, respectively) involved in a disulfide bridge with C^3.25^ in the receptor, and a final tryptophan at the C-terminus of the ECL2 (W186 and W182 for GPR101 and GPR161, respectively). Such tryptophan forms a conserved hydrophobic interaction with Y^6.51^ (Y415 and Y286 in GPR101 and GPR161, respectively) in both receptors, who sits above the toggle switch residue (L412 and W283 in GPR101 and GPR161, respectively) (Fig. 1*C*). Mutating W186^ECL2^ in GPR101 or W182^ECL2^ in GPR161 reduced the basal activity to less than 50% using cAMP assays (57, 58). An additional M184^ECL2^A mutation in GPR101 further reduced the basal activity to less than 10% (56). The conserved set of functional interactions at GPR101 and GPR161 suggests a common activation mechanism between the two receptors despite the low sequence similarity between receptors.

Finally, Type III ECL2 includes GPR55 and GPR61, which contain an ordered loop at the ECL2 devoid of a defined secondary structure (Fig. 1*C*). GPR55 is expressed in adrenal tissue, brain, immune cells, liver and gastrointestinal track and has been proposed to be a potential target for metabolic diseases, Parkinson´s disease, neuropathic pain and inflammatory diseases (73). GPR61 has been associated with biogenic amine receptors and has therapeutic potential to modulate appetite and body weight (74, 75, 76). In GPR55, the central region of ECL2 (residues 170–176) forms an ordered loop that extends into the receptor orthosteric pocket, interacting with transmembrane helices TM2-4 and TM7 (59). However, in this case the structural data was not a strongly supported with mutagenesis and signaling assays. GPR55 is known to be activated by various agonists, and structures of this receptor in complex with such agonists (59, 60, 61) reveals that the ECL2 moves out of the orthosteric ligand-binding pocket in order to accommodate the agonist for further receptor activation. The reason for having constitutive activity and the ability to be ligand-activated is currently unknown (discussed below). In contrast, the ECL2 in GPR61 adopts a different conformation: Only its most C-terminal part penetrates into the ligand-binding pocket, while the N-terminal part, closer to TM4, is disordered and not modeled in the cryo-EM structures. While the ordered C-terminal part is essential for activity since its removal ablates constitutive activity, the loose N-terminal part potentially indicates that the ECL2 is more flexible and tunable by additional modulators, as observed in GPR55, where the loop moves upon ligand binding.

Overall, three different types of in-built agonists have been reported and herein compared. Sequence alignment of ECL2 from all receptors (not shown) does not reveal common conservation patterns except for a conserved cysteine residue which forms a disulfide bridge with C^3.25^ in TM3. This disulfide bridge has been shown to be essential in maintaining the conformation of the ECL2 and activation of GPCRs (77). However, structural and functional data suggest some common activation mechanisms within the different types of in-built agonists (*e.g.* Type II).

## “Constitutively active” receptors due to the presence of ubiquitous agonists

Several oGPCRs displaying constitutive activity in cellular model systems did not show an in-built agonist motif upon structure determination. Instead, they were shown to host a ubiquitous endogenous ligand readily bound to the receptor, normally a lipid derived molecule. In many of these cases, unassigned density visualization of cognate ligands triggered their identification using mass spectrometry and *in cellulo* functional assays. As opposed to receptors that are “born to be active” these receptors are “born to be activated” (78), where the constant presence of such ubiquitous molecules readily modulates receptor function upon insertion in the lipid bilayer. Examples of such receptors include GPR3 (78, 79, 80), GPR6 (81), GPR119 (82), GPR156 (37), GPR84 (83), and GPR174 (14). Additionally, other oGPCRs that also displayed unaccounted densities were GPR12 (39), GPR88 (84), and GPR20 (51). However, in these cases, the identification of such cognate ligands was not pursued.

**Figure 2:**
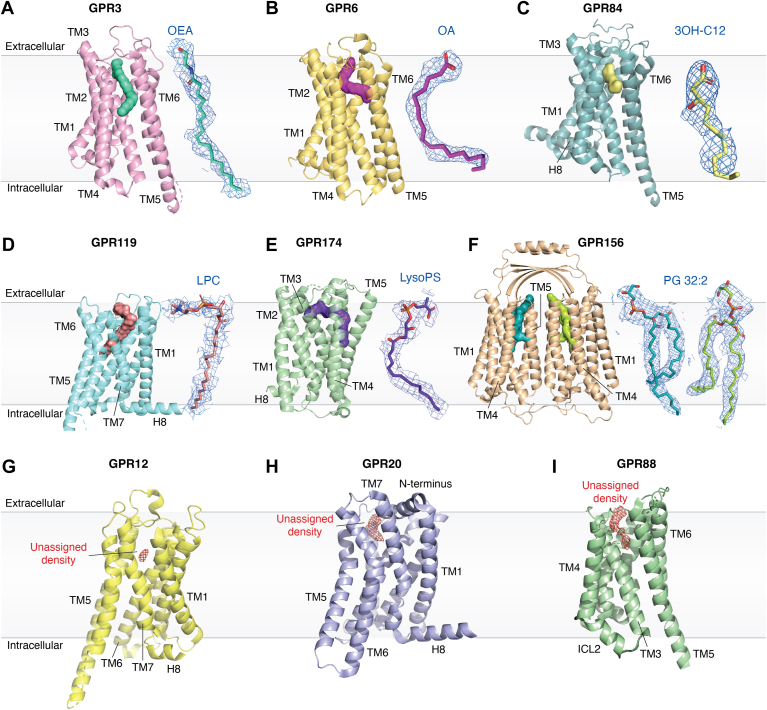
**oGPCRs structures with captured endogenous molecules.***A*, GPR3 bound to oleoylethanolamide (OEA), PDB: 8X2K; (*B*) GPR6 bound to oleic acid (OA), PDB: 8TF5; (*C*) GPR84 bound to 3-hydroxy lauric acid (3-OH-C12), PDB: 8J18; (*D*) GPR119 bound to a lysophospatidylcholine (LPC) (PDB: 7XZ5); (*E*) GPR174 bound to endogenous lysoPS (PDB: 8KH5); (*F*) GPR156 bound to a phosphoglycerol (PG 32:2) (PDB: 8YK0). *G-H*, An unassigned cryo-EM density was detected in the binding pocket of the active receptors GPR12 (PDB: 7Y3G) and GPR88 (PDB: 7WZ4), which likely acts as an agonist. *I*, density in the pocket of the inactive state GPR20 (PDB: 8HS2), suggesting the presence of a molecule potentially acting as an antagonist. All receptors are represented in cartoons with the assigned endogenous ligand displayed as spheres. On the side endogenous ligands, when available, are represented as sticks with their experimental density as *blue* mesh.

In the case of GPR3, GPR6, GPR119, and GPR174, a worm-like density was found occupying the orthosteric binding site (Fig. 2), which after mass spectrometry identification and functional validation using signaling assays, several lipid-like molecules were identified. In most cases, the hydrophilic head of the lipid is exposed to the extracellular side of the receptor, while the aliphatic chain is inserted into a hydrophobic tunnel mainly formed by non-polar residues (Fig. 2). GPR3 and GPR6 are homologous receptors, initially identified to be involved in neurite outgrowth and neuronal cell survival (85). In GPR3, three independent studies simultaneously reported the structural determination of the receptor, identifying an unassigned lipid-like density running through a highly hydrophobic channel that crosses the core of the receptor (78, 79, 80) (Fig. 2*A*). Mass spectrometry identified an enrichment of free fatty acids in one study (80), oleic acid in a second study (78) and oleoylethanolamide in a third study (79). Experiments displaying a higher amount of oleic acid after cold induction further supports the notion that oleic acid is an endogenous modulator for GPR3 (78). Mutation of the large hydrophobic residues in the lipid binding pocket resulted in a significant loss in constitutive activity as measured using cAMP accumulation assays, confirming the constant presence of a lipid molecule in the ligand binding pocket. The study of the phylogenetically related GPR6 receptor presents a tour-de-force using diverse structural, computational, and biophysical approaches aiming to deorphanize the receptor (81). A crystal structure of the GPR6-only and a cryo-EM structure of the GPR6:Gαβγ hetrotrimer revealed a lipid-shaped density running through a channel in the receptor (Fig. 2*B*). Docking a library of metabolites (the Human Metabolome Database, HMDB) (86) ranked lipid molecules as the most likely candidates, which guided efforts in mass spectrometry, functional assays, and co-crystallization studies, all without a clear success. Eventually, manual search and fit of oleic acid into the electron density map combined with computational approaches suggested a potential link between oleic acid and GPR6. This study also highlights one of the challenges in working with caoGPCRs due to the constant binding of ubiquitous lipid molecules, where exogenous molecules in functional assays will display an apparent lack of effect. One approach to overcome such issues is to find mutants that slightly reduce the potency of the agonists (14) (although this is far from trivial) or using different assays that monitor signaling processes that occur close to the receptor (*e.g.* G protein recruitment) since downstream signaling events might readily be saturated with the constant activation of the ubiquitous lipid molecule (14).

GPR119 is a class A oGPCR that was shown to display high basal activity (87) in pancreatic β cells and intestinal enteroendocrine L cells (88, 89) and has been shown to play a role in maintaining glycemic control (88). Cryo-EM structure determination and mass spectrometry drove the identification of four types of lysophosphatidylcholine molecules with different acyl chain lengths (16:1, 18:0, 18:1 and 20:0) with the 18:1 being the most abundant type (82). Coordinates for the 18:1 lysophosphatidylcholine could be modeled into the cryo-EM density (Fig. 2*D*), and its agonistic role was further confirmed and dissected using cellular functional assays. These assays revealed that mutations of the hydrophilic residues interacting with the lipid head group and mutation of large hydrophobic residues interacting with the lipid tail resulted in a significant decrease in basal activity. A similar approach was performed for GPR174, a receptor expressed in lymphoid organs and assumed to have an immunological role (90), where lysophosphatidylserine (16:0 to 20:1 as most abundant lysoPS) was identified. LysoPS bound at GPR174 in a L-shape conformation with the headgroup in the orthosteric site and the hydrophobic tail protruding out of the pocket between TM4 and TM5 (Fig. 2*E*). LysoPS is deeply embedded in the GPR174 core, covered by the ECL2 loop which acts like a lid. In the case of GPR84, a receptor found in immune cells and suggested to have a proinflammatory role (91), an attempt to understand the receptor in the apo form triggered the discovery of an identified density. In this case, mass spectrometry did not result in a clear identification, so functional testing of lipids molecules that could fit the cryo-EM map yielded the identification of a medium chain fatty acid (3-OH C12) as an ubiquitously bound agonist (Fig. 2*C*) (83). In a similar manner as in previous lipid receptors, mutating polar residues interacting with the lipid head group or the large hydrophobic residues interacting with the lipid tail group resulted in a significant decrease in cAMP production.

Finally, GPR156, a dimeric class C orphan GPCR with high constitutive activity present in auditory hair cells (12, 37) showed a phospholipid bound at the extracellular region of each protomer with the phospholipid head group bound between TM5 and TM6 (Fig. 2*F*). Mass spectrometry identified phosphatidylcholine (PC) and phosphatidylethanolamine (PE), being able to identify PC 34:1 (16:1/18:1) as the major species bound to the receptor. Insertion of GPR156 in peptidics containing specific phospholipid compositions confirmed PE/PC as partial agonists while it identified phosphatidylglycerol (PG) as a full agonist of the receptor using a GTP turnover assay. Additional mutations at the lipid binding site and functional readout using cellular BRET2 assays confirmed the location of the ligand binding site. From such studies it was concluded that the highly abundant PC/PE (which account for ∼60% of lipids in the plasma membrane) would ubiquitously activate the receptor, with PG fully activating the receptor when present, in an environment specific manner (normally at < 1% in the plasma membrane). Although a lipid-mediated activation mechanism was proposed using AlphaFold structure prediction, it is still to be determined how the lipid activates GPR156. It is unknown whether the ability to bind PC/PE has been evolved to tune PG binding (*i.e.* only PG concentrations above a threshold would displace PC/PE), or whether the basal activation achieved by PC/PE binding would readily play a functional role.

Additionally, several structural studies have shown the presence of unassigned cryo-EM density in a binding pocket within the receptor. These examples include GPR12 (39), GPR88 (84) and GPR20 (51) (Fig. 2, *G*–*I*). In the case of GPR12, a homologous receptor to GPR3 and GPR6, a small and weak density was found in the ligand binding site (Fig. 2*G*). Such density was too weak to lead to a cognate ligand identification, but parallel studies in the homologous receptor GPR3, identified free fatty acids as agonists for GPR12 (78). In the case of GPR88, a receptor with expression in various tissues but mostly studied for its role in cognitive processes and motor control (92), (1R, 2R)-2-PCCA was thought to be an orthosteric agonist. Upon structure determination, such agonist was discovered to be at an allosteric pocket between TM5 and TM6, while an unexpected cryo-EM density was found at the orthosteric site, within a hydrophobic tunnel similar to the one observed in GPR3 (Fig. 2*I*). Finally, a report on GPR20 presented cryo-EM structures of the receptor in the G_i_-coupled and transducer-free forms (51). An unidentified density was observed in an extracellular pocket when the structure was determined in the inactive state but not present when the structure was determined in the active G protein coupled state (Fig. 2*H*). Such density potentially indicates the presence of a ligand that binds and stabilizes the inactive state, and hence acts as an inverse agonist, negatively modulating the function of receptor. Such hypothesis requires further testing but would pose a negative modulation of receptor function, in contrast to the positive modulation in the canonical signal transduction.

Overall, the new lipid receptors identified are not significantly different in structure or mechanism to the more established lipid receptors such S1P or LPA receptors. However, it is the constant presence of the lipid signaling molecule what has made challenging their deorphanization. Hence, the inclusion of structural determination is particularly well-suited to help identify ubiquitous ligands that are readily bound to the receptor and is enhancing the rate at which new oGPCRs and cognate ligands are being described. The visualization of endogenous ligands through structure determination poses strong evidence for receptor-ligand interaction and contributes to eliminating controversies of cognate agonist assignment arising from functional assays.

## Physiological and therapeutic regulation of caoGPCRs

It is now clear that GPCRs can harbor a variety of in-built tethered agonists within their sequences that can confer constitutive activity (“born to be active”) as well as having ubiquitous modulators that will constantly enhance their basal activity (“born to be activated”). A major question that remains is, how is the function of these receptors physiologically regulated?

First, alternative regulatory mechanisms can modulate caoGPCRs of both types, *e.g.* it has been reported that GPR3 activity is regulated by controlling its expression rather than the activity of the receptor itself. It was found that its expression in adipocytes was induced by low body temperatures, which readily started signaling to promote lipolysis (93). In this case, signaling is terminated upon translation termination and receptor desensitization by canonical arrestin-mediated pathways. Alternatively, some receptors could be regulated *via* endogenous negative modulators instead of endogenous agonists, when spatiotemporally controlled inactivation of the receptor is required. GPR20 could be one of those cases since an additional cryo-EM density was observed only in the inactive conformation (51), however further research is required. Additionally, GPR161’s constitutive activity serves to inhibit Shh signaling and embryonic development, this only occurs upon being transported to cilia by the Tulp3/IFT-A complex, suggesting that subcellular localization plays a key regulatory role (70).

**Figure 3:**
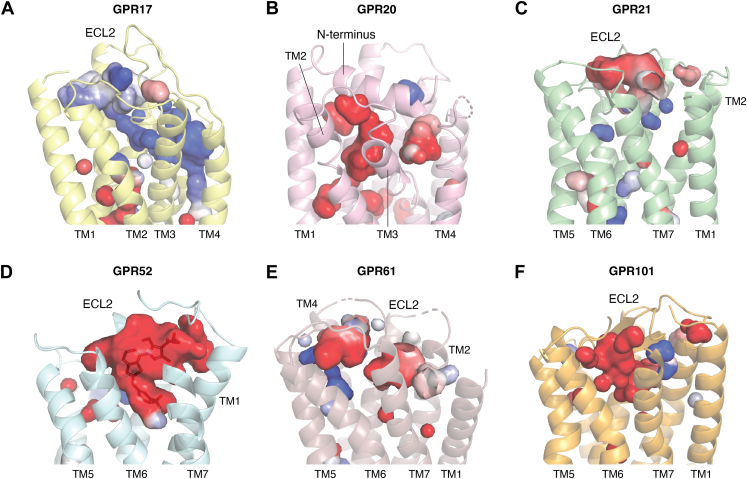
**Additional pockets and cavities of constitutively active oGPCRs.** Pockets and cavities of (*A*) GPR17 (PDB:7Y89); (*B*) GPR20 (PDB:8HSC); (*C*) GPR21 (PDB:8HJ1); (*D*) GPR52 (PDB:6LI3); (*E*) GPR61 (PDB:8KGK); and (*F*) GPR101 (PDB:8W8Q) are shown in surface colored by electrostatic potential. The cavities are obtained by rolling a sphere with the size of a water molecule over and within the protein, as performed in Pymol (http://www.pymol.org/pymol). Major cavities are observed around the orthosteric site despite being occupied by in-built agonists, suggesting the binding of potential endogenous modulators. GPR52 (*D*) shows a modulator bound to a side pocket and interacting with the ECL2 as present in PDB 6LI3. All receptors are represented in cartoons with cavities displayed as surface.

Second, it cannot be discarded that self-activated caoGPCRs have additional modulators further tuning receptor activity. In fact, several evidences suggest that additional molecules could act as agonists or antagonists in caoGPCRs with in-built agonists: (i) most of these receptors have classical activation motifs including NPxxY, PIF, a form of the DRY motif, and a toggle switch residue, all of which are hallmarks in ligand-mediated signaling (94); (ii) all oGPCRs with a tethered agonist but GPR161, also display a pocket for a potential ligand that could further modulate receptor activity on top of the basal activation. Figure 3 shows the calculated cavities in caoGPCRs with in-built agonists, showing a major pocket at the extracellular side. Notably, the pockets are in all cases formed, in part, by the in-built agonist itself, providing the chance for ligands to modulate the in-built agonist conformation or activity. Synthetic molecules binding to such a pocket in GPR52 further activate the receptor and are currently in clinical trials for the treatment of schizophrenia (62). Hence further activation of the receptor is certainly possible, and endogenous molecules could use this pocket to tune receptor activity. Furthermore, the nature of the side pocket varies between receptors, displaying different physicochemical properties in GPR52/GPR21 and GPR17, highlighting the potential variability like the ligands for each receptor (Fig. 3).

Although GPR161 does not seem to have an additional pocket at the extracellular region, it has been shown to require sterol-like molecules binding in a pocket between TM6 and TM7 for it to display constitutive activity (58). Finally, the activating phospholipids in GPR156 might just act as allosteric modulators of yet another cognate orthosteric agonist, since the equivalent phospholipid site of GPR156 in GABA_B_ and mGluR2 is occupied by a negative allosteric modulator and a positive allosteric modulator, respectively. It is yet to be determined whether the extracellular domain of this Class C receptor harbors additional binding sites for yet unidentified endogenous modulators.

If additional ligands are involved in the regulation of these oGPCRs, the question remains as to why they require constitutive signaling if they are going to be ligand-regulated? In a highly speculative manner, one possibility involves the allosteric nature of G protein coupling, in which there is an increase in agonist affinity at the extracellular pocket upon G protein coupling (95), the high basal activity could help enhance the sensitivity of the receptor for their agonists, being able to detect low abundance metabolites or tuning the sensibility of the receptor according to Gαβγ proteins levels. Such high basal activity could also perform a role of inside-out signaling, similar to integrins (96), that would sense/sequester ligands when the right intracellular transducers are accessible. In support of that, nuclear magnetic resonance experiments on GPR52 show an opening and enlargement of the extracellular side pocket upon G protein coupling (97), promoting the sensing of new molecules. Additionally, a cryo-EM structure of GPR61 in the inactive state displayed a fully disordered ECL2 (98), which no longer binds into the orthosteric binding pocket as in the G protein-coupled form, suggesting the reshaping of the extracellular ligand binding site upon G protein coupling.

While GPCR signaling is traditionally viewed as triggering specific cellular responses through precisely timed and localized pathway activation, these receptors may also play alternative, complementary roles. Rather than directly initiating a response, they can influence how responsive a cell is overall. This can happen by adjusting the baseline activity (tonus) of certain G protein pathways or by fine-tuning nearby receptor signaling by depleting specific G proteins. For example, GPR88 has been shown to dampen the signaling of opioid and other GPCRs in the striatum in this way (99).

Finally, additional but poorly understood regulatory mechanisms seem to exist in caoGPCRs. The N-terminus in GPR3 and GPR12 seems to have an essential role in the constitutive activity of the receptors since its deletion yields both receptors mostly inactive (39, 93). In the case of GPR3, even exogenous addition of the N-terminal peptide can rescue its high basal activity (93). However, in none of the cryo-EM structures was the N-terminus ordered or seemed to contribute to ligand binding. Interestingly, for the related GPR6, the N-terminus was not found to be essential for activity (81). It is still to be determined how the N-terminus of such receptors impacts the basal activity of these oGPCRs.

Therapeutically speaking, constitutively active receptors with in-built agonist and bound lipid molecules uncovers new routes to modulate receptors and generate new research tools and therapeutic molecules. At least for some oGPCRs with in-built agonists such as GPR21, GPR52 and GPR101, synthetic modulators have been developed that bind to the extracellular pocket and can further activate the receptor. It is still to be seen whether this pocket is the orthosteric pocket for yet unidentified endogenous modulators, and whether it has similar functional versatility as found in canonical GPCRs, where antagonism, inverse agonism and biased agonism can be achieved at this same pocket. Similarly, one could expect that similar modulators can be developed and used for clinical applications for the other caoGPCRs with different in-built agonists where a pocket has also been identified. Additionally, alternative approaches such as intracellular antagonists can be appropriate for oGPCRs with in-built agonists or tightly bound lipids. Such antagonist has been already described for GPR61(100). Furthermore, it seems that the Type III in-built ECL2 agonist in GPR61 and GPR55 is in equilibrium between a bound activating conformation and a disordered/ligand bound conformation, and hence there would be a chance to trap each state with exogenous molecules to stabilize the bound form or to sequester the ECL2 in a non-bound conformation. This could be achieved using antibodies (100), nanobodies (101) or computationally designed binders (102), somehow mimicking the regulation of protease-activated receptors and adhesion GPCRs, as well as peptide mimetics of the in-built agonist with antagonistic or biased agonism effects. It remains to be seen whether other ECL2 and N-terminal in-built agonist are also in equilibrium and therefore available for such regulation, or whether different types of in-built agonists are easier to regulate than others, *e.g.* tightly bound Type I (GPR52-like) is potentially less likely to be fully dissociated at any moment than the partially disordered loop in Type III (GPR61-like).

## Discussion and conclusions

Orphan GPCRs are a source of untapped potential to understand new physiology and generate new therapeutics, however their poor understanding slows progress in this regard. Over the recent years, structural determination of oGPCRs has allowed the discovery of new modalities of constitutively active receptors that include in-built agonists at the ECL2 and the N-terminus, as well as triggering the identification of cognate agonists with the observation of unknown densities at the orthosteric binding site. Among caoGPCRs with true constitutive activity, three different modes of ECL2 (I - α-helix, II - β-sheet, and III - loop) and an N-terminal mode have been described. Additional modes of in-built agonism could be present in other oGPCRs; however, this classification based on the ECL2 secondary structure attempts to set a base for their study and comparisons and can be further modified as new knowledge appears. This classification can also be useful for further studies using structure prediction using LLM-based approaches such as AlphaFold3 (103). Indeed, AlphaFold is able to predict the most recent structures of in-built agonists presented in this review as well as predict similar Type II β-sheet ECL2 for oGPCRs without an experimental structure, such as GPR26 or GPR78 (predictions available in GPCRdb (104) or Uniprot (105)). Additionally, the structural information will open new routes for receptor deorphanization following computational approaches to deorphanize receptors or to develop surrogate ligands that can be used to interrogate the receptor's physiological role (71, 106, 107).

Based on current data, the ECL2 appears to be the most versatile structural element, diverging to generate different ways to promote self-activation. In classical GPCRs, the ECL2 has a role in capping the binding site, where it has also been implicated in ligand-mediated functional bias (108). It is also intriguing how the N-termini of GPR3 and GPR12 are required for receptor activity despite lacking an ordered interaction in any available cryo-EM structures. A potential explanation is that they engage in a transient interaction in a specific moment of the signaling cycle, or that they mediate interactions in trans with a nearby receptor (*e.g.* in GPCR homodimers). However, a recent structure of GPR3 in a dimeric form did not reveal any ordered N-terminal regions (109).

The physiological regulation and role of constitutively active receptors remains unresolved. Future work must be done to understand whether the only role of the constitutive activity is to activate a signaling cascade, or whether it would fulfil alternative roles affecting the cellular response or the signaling of nearby receptors. In certain cases, a specific regulatory role has been linked to the receptor activity, as is the case for GPR3, where transcriptional regulation has been proposed to regulate receptor signaling. In any case, many oGPCRs with in-built agonists also exhibit additional ligand-binding pockets, allowing further modulation by synthetic compounds. This is the case even when self-activation is described to be comparable to that of classical receptors activated by endogenous agonists. Hence, classical ligand-dependent functions might co-exist with alternative roles.

Additionally, the possibility of the endogenous ligand to be an antagonist (rather than an agonist) has been discussed for long time, and the identification of an unassigned density, exclusively in the inactive state structure of GPR20, can potentially realize this hypothesis. In any case, the question remains whether additional endogenous ligands exist for these receptors and, hence, how long they should be considered orphan receptors?

New receptor-ligand pairings are a non-trivial task, and the IUPHAR has established a set of recommendations for their critical assignment (110). The potency of the proposed cognate ligand should be in line with the physiological concentration of the modulator *in vivo*, and the pairing must be reported by at least two refereed reports (and no contradictory publication) displaying different orthogonal functional assays (*e.g.* signaling and more direct radioligand binding). At times, contradictory functional reports between different laboratories are a major issue (11) and can originate from artifacts of different assays, or from using different cell lines that could have unknown auxiliary elements required for receptor signaling. In this context, structural determination of receptor-ligand pairs poses a strong proof of binding and regulation, which is less prone to artefacts when compared to monitoring downstream signaling cascades. These studies usually combine structural determination with mutagenesis and functional data, which provides strong proof of receptor modulation. Of course, structural determination is only a proof of receptor-ligand interaction, and further studies to validate the interaction and functional modulation *in vivo* must be performed. Also, special care must be taken since structural information is generally obtained using dominant negative Gαβγ heterotrimers which are not able to exchange GDP for GTP and hence trap the receptor in the fully activated state (111). Therefore, it cannot be excluded that receptors with in-built agonists are activated by endogenous agonists, but such agonists were lost during protein purification with the dominant negative Gαβγ holding the complex in an active state. Such a situation could have hypothetically occurred in the case of GPR12, since the homologous GPR3 and GPR6 receptors have been co-purified with endogenous lipids. Additionally, the majority of identified ligands are lipid-derived molecules that originate from the organism where the receptor was overexpressed for structural determination (*e.g.* TnI of Sf9 insect cells). This could lead to the identification of non-native pairings, and careful validation should be performed.

On the contrary, such dominant negative Gαβγ heterotrimers might pose advantages in designing new deorphanization strategies (104). Based on the intrinsic allosteric nature of GPCRs, where coupling of G proteins to the receptors enhances agonist binding (95), using pure fusion GPCRs with dominant negative Gα proteins can serve as a powerful tool to “fish out” low-abundance endogenous modulators in native tissues. This has been somehow performed with GPCR-MiniG protein fusion constructs for such purposes in one of the studies (14).

Overall, high-resolution information originating from structural biology is key in understanding macromolecular functional mechanisms as well as to rationally design therapeutics in the forms of small molecules, peptides and proteins such as nanobodies (101), specially with the recent advances in protein design through artificial intelligence (112). The incorporation of structural biology to the study of oGPCRs is accelerating receptor-cognate ligand pairings and uncovering new modes of receptor regulation. Their understanding will open new ways to regulate receptors with biotechnological applications as well as assisting in the rational design of new therapeutics. With an increasing pace in oGPCR structural determination, the next years promise to be exciting times, with the full impact still to be seen.

## Conflict of interest

The authors declare that they have no conflicts of interest with the contents of this article.
